# Psychology of Malebranche
*"Œuvres de Malebranche." Paris, 1842.


**Published:** 1856-10-01

**Authors:** 


### Art. VIII.—PSYCHOLOGY OF MALEBRANCHE.*

BY PROFESSOR HOPPUS, LL.D.

Father Malebranche ranks among the profoundest thinkers which the school of Descartes has produced. With a somewhat daring imagination for a philosopher, and a tendency to mysticism in his speculations; he was nevertheless an acute, analytic metaphysician, as well as a devout Christian moralist, jealously attempting to guard those of his doctrines which seemed to others most to lean towards fatalism from abuse. He was born at Paris, in 1638, his father being one of the royal secretaries. In consequence of his personal malformation, he had a domestic education, until he was of an age to enter the college De la Marche, with a view to holy orders; whence he passed to the far-famed Sorbonne for his theology. Having refused a canonry at Notre-Dame, he entered the Oratory, in 1660. He now devoted himself much to ecclesiastical history, and read the principal Greek writers on this subject. Richard Simon next drew him to the study of Hebrew; but he had not yet found the line of pursuit which was most deeply to engage his mind. His passion was destined to be for psychological inquiry and original thinking; and he despised the merely historical knowledge of languages, and of the opinions of other men, and was anxious to discover truth, if possible, for himself. It is remarkable, considering his imaginative powers and his enthusiastic temperament, that he never could read ten lines of poetry without disgust. In his studies, his habits were quite those of the recluse; and he meditated with darkened windows, that he might more readily retire within himself; though his manners are said to have been simple and modest, cheerful and complaisant.

Being once in a bookseller's shop, a copy of Descartes' "Traité de l'Homme" was accidentally handed to him—an unfinished posthumous work by no means ranking with the most celebrated pieces of that great philosopher. It made an extraordinary impression, however, on the ardent mind of Malebranche. He found a new world instantly opening to his contemplation, a science of man which he had never before dreamed of. His admiration for Descartes, and the simple child-like docility of that great philosopher as an inquirer after truth, was unbounded. He was delighted with the freedom and independence of thought which characterized his book; nor was he less pleased with its author's avoidance of everything that could tend directly to clash with religious faith. As he proceeded with the perusal, his excitement was so great that it brought on violent palpitations of the

* "Œuvres de Malebranche." Paris, 1842.

heart, and he was obliged repeatedly to lay the book aside. Henceforth neither Greek nor Hebrew, nor ecclesiastical history, had any charms for him; and he devoted himself without reserve to the study of the new philosophy, which was so complete an innovation on the scholasticism that had hitherto reigned—one grand object of which had been to solve all questions, and get over all difficulties, by finding out methods of bringing them under the dogmas of Aristotle, or at least reconciling them with his opinions, so far as these could be made out. Malebranche at once became an ardent disciple of his master, regarding observation as the basis of philosophy, and rational evidence as the rule of its conclusions. He soon appropriated Descartes' entire doctrines, and zealously declared that if his works were by any chance lost, he would do his best to re-establish them. In theology, however, especially so far as regarded the doctrines of providence and grace, he was decidedly a disciple of St. Augustine.

The first fruit of Malebranche's enthusiasm for the new method of philosophical study, was his *Recherche de la Verité* published in 1674; a book which had prodigious success, passing through many editions, and being translated into several languages. The author here points out, in an exact method, the sources of error in the search after truth to which we are liable, from our senses, imagination, inclinations, and passions; and he then treats of the remedies which ought to be applied. All the other publications of Malebranche may be regarded as little more than the development of this work; and his grand aim, throughout, is to show the agreement of the main principles of Descartes* with religion, and their bearings on the illustration of nature and grace. The acuteness, originality, and fertility of invention with which this book abounds, procured for it the highest eulogy. His mystical theory of our *vision of all things in God*, however, subjected it to attacks on all sides; and some remonstrated strongly against the tendency of his doctrine respecting the Divine agency to absorb all subordinate and secondary causes, and along with them man's responsibility.

This first work of our author was greatly altered in the successive editions. He adhered, however, to the theory that the will is the source of error in man, not the cognitive faculties; for the will, he says, guides the formation of our conclusions from the objects presented to us. We know, for instance, that we feel warmth or see light; and here we are not deceived, but only when the will chooses to hold that the light or the warmth exists in the object without. But as sensation gives us pleasure or pain, which chiefly move the will, sensation becomes the main remote cause of error. Hence the false ethical systems which

* For our review of Descartes, see the Number for January, 1855.

make pleasure the highest good; whereas the only true and real good is God himself, whom we can know only by the pure reason or intellect. But how can man know anything of the relations subsisting between mind and matter in the universe around him, since these two natures are so diverse from each other? He can only know them, each and both, in God, and by means of God's ever-acting agency. In this way, alone, is man freed from a life of hopeless and never-ending delusions. Man has ideas, indeed, as his consciousness tells him: but his ideas do not guarantee to him the existence of the objects around him; for imagination also presents ideas, many of which are mere chimeras which never do or can exist. Some of our ideas are internal, or thoughts, strictly so called—modifications of the thinking soul only; other ideas relate to objects which we believe are external to us. These objects are material or spiritual. Material objects can only be perceived mediately, because they are extended, and are not homogeneous with the soul, and have no natural means of community with it: but external spiritual objects may be perceived mediately by ideas, though imperfectly, as well as immediately and clearly, in the Divine vision.

Malebranche wholly rejects the ancient Peripatetic idealism of films, effluxes, species, or phantasms (*tenuia rerum simulacra*) of the shape of bodies, and perpetually flowing off from them to our organs of sense—an inconceivable hypothesis; for what is the image and shape of a sound or odour? He equally opposes whatever glimpse may be found in the Grecian schools of a doctrine analogous to the egotistical idealism by which the name of Fichte is remarkable in later times, according to which the mind spins out from itself the whole web of its own ideas, and creates the entire universe of mind and matter for itself. Our author's argument for rejecting the principle of *innate ideas* would not be admitted by Descartes or Leibnitz, who supposed that the ideas they termed *innate* were limited in number. Malebranche argues against these ideas, on the ground that the "number of ideas which the mind may entertain is potentially infinite, and we cannot suppose, without absurdity, that an infinity of ideas has been originally furnished to our minds." Now, be the doctrine of innate ideas, in any sense of it, right or wrong, no one but Malebranche ever imagined that in order to exist at all, they must be infinite in number. As to the commonly-received notion, that in order to have available perceptions of the objects of sense and thought, the soul only requires its present faculties, and their just development and use—this our author regards as a profane theory, which does nothing less than make man "equal to God," who alone is able to take cognisance of the objects of knowledge, by means of his own proper resources and energies. As the Divine Being, therefore, necessarily has in His infinite mind the ideas of all things, He alone is man's "intelligible world:" all His works must be seen and known *in Himself.* The result is, that not matter only, but even created mind, has only, in and for itself, a sort of passive activity: all its operations and agencies are only secondary; it cannot possibly, from its very nature, have any independent power or spontaneity conferred on it: it can truly originate nothing.

In order to render his views, which some thought obscure, and others (what was still worse for an ecclesiastic) heterodox, more adapted to the popular mind, and to obviate the idea that any of them were not in accordance with the doctrines of the Church, he published his *Conversations Chrétiennes* in 1677, at the instance of the Duc de Chevreuse. In 1680 appeared the *Traité de la Nature et la Grace,* occasioned by a controversy with Arnauld on the subject, in which Bossuet also took part against Malebranche, but which ended, like many other such disputes, without result. In the same year he published his *Méditations Chrétiennes et Métaphysiques,* a dialogue between the Word (Λόγος) and the author, with a view to throw further light on the above Treatise; for he held that the "eternal Word is the universal reason of spirits:" "Comme je suis convaincu que le Verbe éternel est la *Raison* universelle des esprits, je crois devoir le faire parler comme le véritable *Maître.*"* In the year 1682 the *Traité de Morale* followed, in which Malebranche endeavours to derive all human duty from his own philosophical principles as perfectly coinciding with Christianity, and to prove, in his own way, the union of all spirits with the Divinity. The *Entretiens sur la Métaphysique et sur la Religion* were published in 1687, and some have pronounced this work to be the author's *chef-d'œuvre.* Its tone is, as usual with Malebranche, elevated, solemn, and devout; and it is written in a finished and attractive style of dialogue ,which Plato might have envied: our author himself, however, preferred the "Méditations." Both works are in his best manner; but the "Entretiens" may be regarded as furnishing a clear, animated compend of his entire philosophy. Being accused by Régis of abetting the ethical system of Epicurus, and by Father Lamy, on the other hand, of advocating an exclusively disinterested love of God, he published his brief tract entitled *Traité de l'Amour de Dieu,* in 1697, in which he maintains a medium between extremes; and his book conciliated Bossuet, and was praised at Rome. From some of his works having found their way to China, probably in the hands of Catholic missionaries, he composed his short dialogue, *Entretien d'un Philosophe Chrétien, et d'un Philosophe Chinois,*

* "Médit. Chrét.," *Avertissement.*

*sur l'Existence de Dieu*; a work which procured him blame from some quarters for his assertion that the Chinese philosophy was atheistic, while the retort of symbolizing with Spinozism was made upon himself for his mystical views regarding the "extension of the infinite."*

We must not attempt to enumerate the other writings of our author, epistolary and polemical. His replies in the controversy with Arnauld were collected and published in four small volumes, at Paris, in 1709. We may add that he was a geometrician and natural philosopher, as well as metaphysician; and he was received as an honorary member of the Academy of Sciences in 1699. A small piece of his is extant, entitled *Traité de la Communication du Mouvement*, in which he recants his statement in the "Recherche," that the same quantity of motion is always preserved in nature; and he added some physical remarks on the general system of the universe. Though our author's philosophy has not by any means preserved the reputation which it had when it first became known, (in a sphere where *new lights* have mostly been attractive till they were neglected for *newer*,) his metaphysical talent, the beauty of his style, the sincerity of his aims, the seriousness of his tone, and the elevation of his thoughts, will always insure to his writings a considerable amount of well-deserved attention. Occupied as he was with abstract studies, he appears to have possessed an eminently pious mind, and to have been very punctual in all his duties. On a visit which he once paid to the great Condé, at Chantilly, the attendants remarked that he "spoke more of God in those three days than the prince's confessor did in ten years." His company was much courted, notwithstanding his great love of solitude, and a certain irritability which crept on him in his maturer years. King James II. sought an interview with him; and he was visited by Leibnitz and Berkeley. In a conversation with the latter, the discussion became so warm, and Malebranche was so excited, that his feeble and aged frame could not sustain the shock, which hastened his end. Though naturally of a weak constitution, with frequent ailments, his great temperance and regimen prolonged his life to the age of 77, when he died, in the year 1715.

In the strict chronological order, Spinoza comes before Malebranche; but in the order of development the philosopher of the Oratory stands nearer to Descartes, the original founder of the first school of Continental idealism. Malebranche's advance from

* "Oui, sans doute, l'étendue, celle que vous apercevez immédiatement et directement, l'étendue intelligible, est éternelle, nécessaire, infinie. Car c'est l'idée ou l'archétype de l'étendue créée, que nous apercevons immédiatement; et cette idée est l'essence éternelle de Dieu même, en tant que relative à l'étendue matérielle." *Entretien*, etc.

the Cartesian point of departure, though well defined, fell short, in its extent and boldness, of that of Spinoza, whose system amounted decidedly to a theoretic Pantheism. With Descartes, Malebranche sought for truth in our intuitive and rational conviction of certitude; but he looked at this subject in his own mystical way, and what he says on it only presents another phase of his leading doctrine of vision in God. He maintains that there is, *first*, an "essential reason" common to all intelligences—an eternal light superior to our minds, which contains in itself all the principles of the sciences and the arts, morals and laws—a reason supreme and necessarily existing. This comes very near to, or is rather identical with, the doctrine of "impersonal reason" in the Eclectic school of France; and it would seem of necessity to mean one of two things, each of which is inconceivable. Is reason, we may ask, *ours*, or is it not? If it be ours, how can it be *impersonal*—no part of us—beyond our minds? Is it, then, like the light of heaven, by which we see, but which is not dependent on our senses? Such an analogy, we repeat, is inconceivable as applied to mind. Reason, as human, must be individual; its individuality is no more set aside by its uniformity in the race, than the individuality of digestion is overthrown by its being common to all mankind. But is reason God's, and not man's? (of course, we are here speaking of the "essential reason"—the "eternal light," which, according to Malebranche, contains all the principles of our rational knowledge:) if so, then how can man, as man, attain to truth? Figuratively and poetically, we may speak in this strain of the superlative excellence of reason very well; but our author meant it for philosophy. *Secondly*, according to Malebranche, there is a natural reason common to all men, the gift of the Creator, and analogous to the eye of the body: it is, in fact, the intellectual eye which contemplates the light of the supreme reason. And, *thirdly*, there is in the world a sort of arbitrary or factitious reason, purely human, which every man makes for himself, to substitute in the place of the universal reason, and more particularly in all matters which relate to his own conduct. From this last form of reason, according to Malebranche, moral evil arises; for error and moral evil are the consequence of the natural reason not being properly turned to the contemplation of the eternal reason.* Many of our author's remarks on this subject, in various parts of his works, are exquisitely beautiful and sublimely devout; but when viewed psychologically and analytically, and as offering a system of the mind, they are of too mystical a cast to approve themselves to our understanding. If,

* "Méditations Chrétiennes," etc., *passim*.

however, we ought not to say, with some of his admirers, that he was the "profoundest thinker" whom France has produced, we may well admit that he is one of the finest writers who ever graced the French language. Diderot said, "One single page of Locke contains more truth than all that Malebranche has penned; but one line of Malebranche is marked by greater subtilty, more imagination, acuteness, and genius, than Locke's whole 'Essay.'"

As a priest, Malebranche was more called than even his cautious master Descartes to avoid giving offence to the Church, and he constantly aims at illustrating theology by metaphysics. Though his ideal theory of perception, by maintaining that we "see all things in God" (*nous voyons tout en Dieu*,) rendered the outward universe unnecessary; yet he did not, with Berkeley, deny its existence: for he conceived that the cosmogony of the Mosaic statement, that God, in the beginning, "created the heavens and the earth," obliged him to admit the real existence of matter; while Berkeley, on the other hand, does not appear to have felt any difficulty in understanding the language of the Pentateuch in regard to the creation of the world as merely accommodated to the general impressions of mankind, and the natural tendency of their sensible ideas, and as not at all affecting the metaphysical question respecting the independent reality of matter. In many instances, however, we see in Malebranche, as Dugald Stewart has observed, "a remarkable boldness and freedom of inquiry, setting at nought those human authorities which have so much weight with men of unenlightened erudition, and sturdily opposing his own reason to the most inveterate prejudices of his age. His disbelief in the reality of sorcery, which seems to have been complete, affords a decisive proof of the soundness of his judgment when he conceived himself to have any latitude in exercising it." In fact, the Congregation of the Oratory at Paris was always distinguished not only by the learning, but by the moderation of its members, and was more Jansenist than Jesuit in its complexion. Malebranche himself appears to have had strong misgivings against the persecuting spirit of the Romish Church.

It was not till he had spent about ten years in the study of Descartes' philosophy, that he published his principal work, already noticed, and first announced his main doctrines respecting understanding, or "pure spirit," (of which, our author says, thought is the only essential attribute,) and respecting our "seeing all things in God," which he seeks copiously to prove from Scripture, maintaining that God is the place of spirits, as space is the place of bodies, and that it is therefore certain that the mind of man can see in God those ideas which must exist in the Divine Spirit, representing all created things. The mind can see in God the works of God ; and God allows it to do so, and prefers giving to man this vision in himself and of his divine ideas, to creating an infinite number of ideas in each spirit. The latter alternative is precisely the Berkeleian idealism; but, according to Malebranche, since God is united to us closely by his omnipresence, we as much see his independent ideas with our minds as we see with our eyes bodies around us in space.* It is only thus that we can have ideas of pure truth, unmixed with the delusions which are blended with the operations of sense and imagination, which, together with pure mind (*esprit pur*) or understanding, are the three faculties of the soul. He develops this theory of a kind of theosophic vision, in the third book ; and the whole discussion on this subject, both here and elsewhere, savouring as it does of Platonic mysticism, cannot fail to be interesting to the reader, on account of the deep spirit of devotion with which it is animated. The next two books are on the inclinations and passions, with the errors and evils which they may occasion, and the inquietude of the will in our search after happiness. The last book of the "Recherche" is on *Method*, or the means of strengthening the attention and enlarging the mind, and the proper use of the senses, the imagination, and the passions : rules are added for the discovery of various kinds of truth.

Our author, in the above work, and in all his other pieces, evidently appears in a twofold capacity. He is the commentator on Descartes, reproducing his ideas, not excluding his faults, in a very animated and perspicuous manner; with his master, assuming the material theory of *animal spirits*, and its bearing on memory, imagination, and other faculties of the mind; but, like his master, rejecting the ancient doctrine of *species* emanating from material objects. He was Cartesian in setting out from observation external and internal, and in his doctrine of soul as a thinking, and of body as an extended substance ; and on this subject, like Descartes, he often substituted the language of direct idealism for that of attributive description, by speaking of extension as the essence of body, and of thought as the essence of mind : he was Cartesian in the theory that the *idea* of the infinite (God) proves the *existence* of the infinite, and in holding that the veracity of God is the basis on which our belief in the existence of matter ought to rest; but we must not omit the fact that Descartes held *that* veracity to be involved in the testimony of sense and consciousness merely ; he did not adduce the Mosaic account of the creation, with Malebranche. Our author,

* "Il est certain que l'esprit peut voir ce qu'il y a dans Dieu qui représente les êtres créés . . . l'esprit peut voir en Dieu les ouvrages de Dieu," etc.—*Recherche*, iii. 6.

however, was an original speculator, as well as a disciple of Descartes; and he evidently put forth views which, though their germ may be found in Cartesianism, were such as Descartes himself would have startled at, and have regarded sometimes as altogether untenable, and, at other times, as exaggerations if not caricatures of his own theories. Malebranche's *vision in God*, when regarded as a philosophical tenet, and not as a piece of poetical imagery emanating from a mind filled with devout enthusiasm, is none other than a paradoxical dogma, accompanied as it is with the denial of any power in the mind to have true ideas of its own. Its popularity in France, among numerous disciples, was partly owing to its being supposed to be in harmony with certain views of no less renowned a Christian Father than St. Augustine. Even in England the doctrine was not destitute of countenance; witness Norris's "Essay towards the Theory of the Ideal or Intelligible World," in which the writer is more of a Platonist than Malebranche himself. On the other hand, he was combated with much wit and humour by Antoine Arnauld, in his book "Des vraies et des fausses Idées;" and Locke replied to him in a small treatise among his posthumous works. The prominence which Descartes had given in his speculations to our idea of the infinite, more particularly as involving an ever-efficient interference in sustaining and actuating all the operations of nature, tended to throw into the shade all finite power and action, whether in created intelligences or in the universe of matter. Geulinx of Antwerp, a zealous Cartesian, distinctly maintained that all created agency only marked the *occasions* on which the Creator himself put forth his own causative energy. Malebranche completely endorsed this theory of the dynamics both of matter and mind; and it is obvious that this decidedly pronounced and extended development of the original Cartesian view of the "*concursus*" and "*assistentia*" of the Deity, when once it had assumed its last form of "occasional causes," strongly tended to merge all things, including all mental operations, in the infinite: so that not only our true perceptions, but even our very consciousness itself, could only be depended on, according to Malebranche, in proportion as we see ourselves, and all besides, in God. The way was thus prepared for the pantheistic system of Spinoza.

Descartes had already too much restricted the sphere of the soul or mind to thought: he regarded the thinking being chiefly in the light of an intelligence, not a power. This, at all events, was the point of view which he mainly fixed on in surveying man; and his speculations in reference to human activity, tended to a considerable extent to lower the faculty of volition into mere inclination and desire. Malebranche, who had far more imagination and much less of a tendency to cold logical abstraction than his master, almost merged the creature in the Creator: human power became in his philosophy a vanishing quantity, losing itself in the Divine agency; and even thought itself only possessed validity as reflected from the pure mirror of the Divine essence—not, indeed, itself seen, but exhibiting to the mind of man all the real forms and images of truth. The mode in which substances act on each other has always been a problem in philosophy, and all that we still know of this reciprocity, after ages of speculation, is the fact itself. The difficulty has been supposed to be increased, from the earliest times of philosophy, when the substances are heterogeneous; but while the fact, if we put any trust in our senses, is as indisputable in the latter case as in the former, the solution is not easier in the former than it is in the latter. Malebranche at once denies both the fact and the possibility of any such intercommunication as can be efficient, between created substances, like or unlike. He regards the distinction of body and soul as the basis of all our knowledge of man;* but he maintains that neither matter nor finite mind can produce any effect on either mind or matter. The real abyss which lies between two things apparently so near together as mind and matter, can only be spanned by the Divine intervention; the chasm is bridged over only on the principle of "*occasional causes*," and of Divine grace which comes under them: in other words, the apparent agency of what are termed second causes is not real—these so-called causes are the mere occasions of the putting forth of the Divine power. The will of my soul is not the immediate antecedent to the motion of my body; it is only the occasion on which God himself produces that motion: so of the impression apparently made by an external object on any organ of sense. "For example, when my hand is burned by the fire, it is not the fire which raises the idea of pain, but an opportunity is thus given for the Almighty to produce such an idea in me. And when I wish to move my finger, it is not my soul that moves it; but the Almighty, through the channel of my will, takes occasion to move that portion of my body."†

From the mode in which our author treats of vision in God as the source of all pure knowledge, and as the result of "union with him," it follows that the laws of human reason and thought which, we have evidence, have existed coevally with man, and which we cannot but believe will exist as long as the race is in being, are not to be regarded as constitutional principles of the human mind—they are rather the very intelligence of God himself, of which man partakes. "The Word of God alone,"

* "Entretiens sur la Métaphysique," i. 3.—"Recherche," iv. 2.

† *Ibid.*

repeats Malebranche, "is the universal reason of spirits."* We wish our space would allow of our quoting a number of long extracts on this subject: many might be introduced which are so exquisite in the expression, and in a tone of such impassioned devotion, that they would be well worth the perusal for their own sake. We must be content with introducing a few of them to the reader, agreeing, as we do, with Dugald Stewart's remark, that it is seldom possible to do justice to Malebranche in an English version.† We will, however, translate a passage of a more expository character, illustrative of the subject, and of the author's quieter and more didactic manner, in which the "Word of God, the universal reason of spirits," is introduced as instructing the serious and docile inquirer:

"When thou seest that twice two make four, and that twice two do not make five, thou seest truths; for it is a truth that twice two make four, or that twice two do not make five. But what dost thou see, then, but a relation of equality between twice two and four, or a relation of inequality between twice two and five? Hence truths are only relations, but relations real and intelligible. For if a man were to imagine he saw a relation of equality between twice two and five, or a relation of inequality between twice two and four, he would see a falsehood; he would see a relation which did not exist, or rather he would believe he saw what in fact he does not see.

"Now, all these relations may be reduced to three kinds: relations between created beings, relations between intelligible ideas, and relations between beings and their ideas. But as I include in my substance only purely intelligible ideas, it is only the relations existing between these ideas which are eternal, immutable, necessary truths. The relation of equality between twice two and four is an eternal, immutable, necessary truth; but the relations existing between created beings, or between these beings and their ideas, could not begin before these beings were produced; for there is no relation between things non-existent: a nothing considered as such cannot be double or triple of another nothing, nor can it even be positively equal to it.

"I am thus eternal truth, because I include in myself all necessary truths. I am truth, because there is nothing intelligible out of me: it is not that I pour light on spirits as a quality which enlightens them, but that I discover to them my substance as the truth or the intelligible reality by which they are nourished: it is that I unite

* "Il n'y a que le Verbe de Dieu qui soit la raison universelle des esprits."—*Méditations*, ii.

† "O Dieu, que d'obscurités et de ténèbres dans mon esprit!" etc.—*Méditations*, iv.

"O Jésus! vous m'avez dit que vous êtes l'ordre aussi bien que la vérité," etc.—*Ibid.*

"O mon Jésus! vous êtes la raison universelle des esprits et leur loi inviolable; vous êtes la lumière et la sagesse éternelle; vous êtes l'ordre immuable et nécessaire! Dieu n'éclaire les hommes que par vous, qui êtes son Verbe," etc.—*Méditations*, v.

them directly to myself as to the reason which renders them reasonable: it is that I give myself entirely to every one of them, it is that I pervade them, and that I fill the entire capacity which they have to receive me. But thou art not in a condition to comprehend clearly how I communicate myself to men."*

We will quote one more expository passage on the subject of the nature and office of reason, from the "Traité de la Morale:"

"The *reason* of man is the *Word* or the Wisdom of God himself; for every creature is a particular being, but the reason of man is universal. If my own individual mind were my own reason and my light, my mind would also be the reason of all intelligent beings; for I am sure that my reason enlightens them all. My pain no one can possibly feel but myself, but every one may recognize the truth which I contemplate; so that the pain which I feel is a modification of my own proper substance, but truth is a possession of all spiritual beings. Thus, by the instrumentality of reason, I have, or may have, some society or intercourse with the Deity, and all other intelligent beings; because they all possess something in common with me, namely, reason. This spiritual society consists in a participation of the same intellectual substance of the Word from which all spiritual beings may receive nourishment. In contemplating this divine substance, I am able to see some part of what God thinks; for God sees all truths, and there are some which I cannot perceive. I am able also to discover something of the *will* of the Deity; for He wills nothing but in accordance with a certain order, and this order is not altogether unknown to me. It is certain that God loves things according as they are worthy of love or esteem; and I can discover that there are some things more perfect, more valuable, and consequently more worthy of love than others."†

In regard to matter, Malebranche was as much a cosmothetic idealist, *practically*, as Berkeley himself. He admitted matter, from necessity, believing that its existence was a subject of revelation; but he made no use of it. All that we have really to do with, according to him, are the things which we see in God. He was, in this respect, more Platonic than even Plato himself; who, while he sought to elevate himself and his disciples to the contemplation of the intelligible and archetypal world, as it subsisted in God, did nevertheless admit a certain commerce with sensible things, a certain vision of the sensuous images of the *ideas* which were wholly intellectual and beyond the ken of sense. Malebranche is more restrictive: with him the faculty of perceiving the *Divine* ideas is the only intelligence which man can arrive at. Even when we fancy ourselves looking at the external world, it is only spiritual or non-sensuous *ideas* that we see, and such ideas alone are the objects of knowledge.

* "Méditations," v. 4, 5, 6.

The presence of objects is the occasion only, not the cause of our sensuous impressions. Our ideas are neither derived from matter, nor from the operations of our own minds. Our author thus speaks on the subject, in the first of his "Entretiens sur la Métaphysique,"* which consist of very animated and attractive dialogues, much in the manner of Berkeley. Theodorus, who represents Malebranche, thus addresses Aristus, a learner who is not yet quite able to digest all the mysticism of his able and painstaking instructor:

"You do not follow me, Aristus; your room is, in itself, absolutely invisible. What I see when I look at your room, and survey it from side to side, will be always visible, though your room were destroyed. What do I say!—yes, though your room had even never been built. . . . . I repeat it, Aristus, strictly speaking, your room is not visible. It is not properly your room that I see, when I look at it; for I could see all I now do, even had God destroyed it. The dimensions which I see are immutable, eternal, necessary. These intelligible dimensions occupy no place. But I am afraid of multiplying your difficulties, by telling you too many truths; for you appear to me to be embarrassed enough in distinguishing ideas, which alone are visible by themselves, from the objects which they represent, which are invisible to the mind, because they cannot act upon it, nor be represented to it."

Elsewhere† Theodorus tells Aristus that, "though bodies are invisible by themselves, the sense of colour which we have in us, and even in spite of ourselves, *on occasion* of them, makes us think we see them." Arnauld remarked that, to suppose things to be only visible in God, and consequently that we also see space in Him, is impossible, since the Divine Being is not extended. The reader must judge how far Malebranche threw any further light upon the controversy, when he replied that "intelligible space, which we see in the Divine substance as including it, is only this same substance in so far as representative of material beings, and participiable by them."‡

The theory of *occasional* causes, which, as we have seen, identified all the operations of mind and matter with the direct agency of the Deity, and rendered every movement of the human body, and every human volition, a result carried into execution by an immediate act of the Divine power, easily harmonized with the other grand tenet of Malebranche's transcendental mysticism, which involved not only the whole field of activity, but also that of human perception. It was not in action only that there was contact with the Deity—in the vision which man can have of God, and in Him, there was a revelation to man of everything by a Divine union and illumination; so that

* Section vi.  † "Entretiens," xii. 2.  ‡ *Ibid.* i. 6.

ideas or images which appear to be states of our own minds are really attached to the Divine intellect, in whom we perceive them: our ideas, so far as they are ours, are but ideas of ideas already existing in God, and seen in him. Bayle, in his Dictionary, has well remarked,* that while these views of Malebranche approached to some speculations of the latter Platonists, there is a still closer resemblance between them and the opinions of some of the Hindoos; who, according to Sir William Jones, "believed that the whole creation was rather an *energy* than a *work*, by which the Infinite Mind exhibits to his creatures a set of perceptions, like a wonderful picture or piece of music, always varied yet always uniform." Of course, Malebranche was consistent enough in arguing, as he did, against the reality of the secondary properties of matter, such as smell, taste, and the like; but his reasonings, if fully developed, would equally avail against the primary attributes of resistance and extension. Bayle remarked this consequence, in anticipation of Berkeley. Yet Malebranche held fast to matter as a substance *revealed* on the Divine veracity. This tenet, however, was an isolated part of his system: for having got matter, he may be said not to have known what to do with it. Man had no concern with it in itself, but only through the Deity: our perception (*idée*) of the primary qualities he regarded as a kind of objective intuition of them as manifested in the Divine intellect. His distinction, however, between primary and secondary properties, and between perception and sensation (*sentiment*) is more precise, as the late Sir William Hamilton has remarked, than that of Descartes himself, or any previous metaphysical writer.

All the parts and aspects of our author's theory of *human knowledge* are ruled and pervaded by his fundamental dogma of the theosophic vision. His formal definition of the term *idea*, excepting so far as he distinguishes it from sensation, is vaguely expressed as marking that which is the "immediate object of the mind, and is present to it when it perceives anything."† He further states that the general object of all ideas is the *extension of the infinite*, supersensible, unchangeable, and incommensurable; from the perception of which we obtain an image of everything we perceive either within or without ourselves; and this, he repeats, is truly an insight into God himself. While thus contemplating the extension of the infinite and the supersensible, we do not perceive the very substance of the Deity: we perceive him "only in so far as all created beings partake of his substance." What our minds actually see, our author tells us, is imperfect, while God is perfect. What they perceive is finite, divisible, figured, and individual: but the Deity himself is

* Article "Amelius."

† "Recherche," iii. pt. 2, ch. 1.

"*all beings, but no being in particular.*"* (Here is a decided point of contact with the doctrine of Spinoza.) Further, there is a wide difference, says Malebranche, between that perception of ideas, *i. e.*, modifications of the infinite, which we call *knowing*, and the perception which we have of modifications of ourselves as subject, which is feeling. It is knowledge alone that furnishes objective truth, the senses only afford subjective experience: nevertheless, sense can lead us to a knowledge of truth, if we only remember that the qualities and modes of objects which appeal to our senses merely exhibit the relations of one and the same extension of the infinite to our intellect.† We are here reminded (bating the mysticism) of some of the views which Kant has elaborated in his doctrine of the sensible intuition *(anschauung)* of space. Our author concludes that the source of error lies *partly* in sense, which perceives only what is external, but not what lies concealed under the exterior, which alone is objectively true; *partly* in imagination, which can only survey what is material; and, finally, *partly* in the freedom of our reason itself to follow either the sensuous or the supersensible (rational) mode of perception.‡ Our perception of God is immediate, without image or idea; but bodies are perceived by means of images or ideas in God himself, as modifications of the extension of the infinite. In regard to the vexed question of *innate ideas*—on Malebranche's principles, it is evident that there was really no place for them. They are totally unnecessary, for true knowledge of all kinds is identified with seeing and knowing all in God. The true ideas are in *His* consciousness, primarily, and there man takes cognizance of them.

The student of our author, as he advances in the perusal of his writings, and compares together the reiterations of his fundamental doctrines, in varied forms of expression, will find more and more evidences of a Spinozistic view of the relation between God and his works. Not only does the Divine Being "contain in Himself the perfection of all created things;" not only is He said to comprise within Himself all finite spirits, and to be their *place;* not only is His substance their intelligible (supersensible) world, in which they live and perceive; not only is He the "life and soul" of all spirits, as being intimately united with and present to them: if all this, did it stand alone, might be interpreted as nothing else than the poetic imagery of devotion, we have more—we are told that "all spirits, all souls, and all bodies, subsist as *modifications of the extension of the infinite and the supersensible.*"§

* "Recherche," iii. pt. 2, c. 6. See also "Entretiens."
† "Entretiens." ‡ "Recherche," i. c. 4, 5.
§ "Recherche," ii. c. 6.—"Réponse à M. Régis."—"Conversations Chrétiennes," Dial. iv.

One of the most striking illustrations of the length to which our author went in attaching weight and significancy to the language in which he, as above, describes his views of the relation subsisting between the Deity and his creatures, is found in the remarkable fact that he denies that man can have any independent personal *self-consciousness!* He cannot know himself by reflection—by introverting the mental eye, as it were, and turning it on the operations of the inner man. He can only become conscious of himself by seeing himself as reflected from the mirror of the Divine mind. Descartes had held that our mental constitution was capable of giving us clear and distinct ideas not only of nature around us, but primarily and most convincingly of *ourselves.* Consciousness of the *ego* was, with Descartes, the solid fundamental ground on which man might go forth to the business of reasoning and searching after truth. The consciousness of thought was the irresistible voice within, which announced the most certain of all truths to me—my own existence; and this beyond the possibility of contradiction or even doubt. Malebranche, however—Cartesian as he professed to be—would not admit even the testimony of *consciousness* as an earthly witness: consciousness herself must first have her apotheosis, before she can be credited—the very consciousness of man must be an appendage to the consciousness of God. "We know ourselves by consciousness, or the inward sense which we have of our actions,"* says our author; yet he maintains that it is not consciousness that gives us a true notion of ourselves, or of our being: "We remain unintelligible to ourselves till we see ourselves in God, who presents to us an idea altogether clear of our own existence."†

Of course, human *reason* is not more strictly personal than human consciousness. We have already remarked that it is in Malebranche that we find the element of the doctrine of the *impersonality* of man's reason, as derived through some of the later Germans to the French Eclecticism. "Human reason," says our philosopher, (and he is here speaking of it in its essential nature,) "is the Word and Wisdom of God himself. It is therefore a participation of the Divine substance; and we may, by means of it, see on our part what the Infinite God thinks."‡ "And being thus united to the Deity in intelligence and perception, all good will be revealed to us, and all happiness—in His light: and we may thus will and accomplish some good things, as He wills and accomplishes all that is good."§

Virtue, our author regards as the habitual and predominant love of the immutable order which proceeds from the cognition

* "Recherche," iii. pt. 2, c. 6. † *Ibid.* iii. pt. 3, c. 1.

‡ *Ibid.* iii. pt. 2, c. 6.

§ *Ibid.*—"Traité de la Morale."

of God; and, to attain to it, man must be delivered from the dominion of sense and passion. It was to be expected that his views of human volition should be wholly ruled by his doctrine of *occasionalism* already noticed. Every action, according to him, is properly such that all real exertion of force belongs, not to the creature, but to God: we *will*, God produces—our will precedes the effect, God *causes* it; there is but one cause, for only one being is efficient, that is God. It is true, created beings are made the "*means*" of the Divine action, but only "according to the exigency of the occasional causes." God alone is the true cause of all things that take place. It is evident that Malebranche considered the doctrine of *assistentia* and *concursus*, which we have remarked was propounded by his master Descartes, to be precisely identical with his own; though it was not so prominently brought forward and blended with the whole method. "Creatures," says our author, "are nothing but occasional causes; and this entire world is only a system of occasional causes, as Descartes has correctly taught."* On the doctrine of *final* causes, the disciple differed from his master, who had rejected them as an object of inquiry, or an evidence of Theism. To the mind of Malebranche, they appeared in a different light: all events and all harmonies are to be traced immediately to the Divine intention and will: and he attaches importance to the remark, that "if created spirits could know anything as detached from the Deity, it would follow that they were not formed entirely for the knowledge of him."† God is "the infinitude of space and of thought; bodies and minds have only a passive capacity in him:" occasional causes involve in their very nature the final causes which led to the occasion of their being employed by the great, first, and only true and efficient cause. It is obvious that the doctrine of Malebranche on this subject involves, or rather requires, the whole length of Leibnitz's pre-established harmony.

Descartes had grounded philosophy in God's being and subsistence, as the absolute basis of *à priori* truth; man's own conscious existence leading immediately by a single step (the psychological idea) to the irresistible belief in a Creator. Malebranche followed his master in seeking to arrive at truth by the *à priori* road; though he gave more prominence to the important axiom that the laws of physical nature must be discovered by an inductive logic, a principle which he properly set in a strong light. On the other hand, where Descartes would have reasoned by deduction, Malebranche had recourse to that pure intuition which was the bond that united the finite mind to the absolute and the infinite. "We see, and know, and do all things in God," is the single text of his entire philosophy.

* "Entretiens sur la Métaphysique." † "Recherche," iii. pt. 2, c. 6.

No theologian could go much further than our author in maintaining the doctrine of human impotence, in respect to what is morally good. It was an essential part of his creed; and he carried it to an extent which seems scarcely to leave room for man to be fairly regarded as a being fully accountable for his actions. Not that we would attribute such an intention to Malebranche himself—he is far enough from it: we only speak of the obvious tendency of his theories, and of many of his comments on them. He denies to man all moral power, excepting that of deceiving himself as to what is morally good or evil. Man cannot form even a particular desire of good; he can only have a general apprehension of it. Of course, he cannot really pray to God—his devotional consciousness must itself be a consciousness that can hardly be called his own. His prayer, if we put the usual interpretation on Malebranche's language, would scarcely seem to be different from that of an automaton so constructed as to utter words.* No doubt we are bound to take into account the religious and highly imaginative strain in which he treats all the subjects that come under his notice—we do not wish always to hold him to the letter: after all deductions, however, little scope seems left by his theory for such a rational freedom as is absolutely necessary for realising the idea of man's accountableness.

Nevertheless, like many other sanguine minds, not masters of themselves, and still less of their beloved theories, (Leibnitz eminently for instance,) Malebranche thought he saw the reconciliation of reason and revelation—of philosophy and faith, and the solution of the great and awful mysteries of moral evil, providence, grace, and human destiny, in the sublime ecstatic vision of the eternal reason, light, and love, to which man may attain in this world, in proportion as he is freed from the illusions of that "arbitrary and factitious reason" which he substitutes for that which is divine. Like Leibnitz, wishing to explain, if not to comprehend everything, Malebranche also had his *optimism*, though not by name. He does not speak, indeed, of a "best possible world," but of a world "constructed according to the most simple and general principles." He asks whether it would be well for evil not to have existed, on condition of a greater complication of means and laws? and he decides the question in the negative. God desires good by the most simple possible means, and general laws are more of a good than evil is of an *evil*. The stability of the whole system depends on the performance of the Divine volitions, that is, of the Divine laws: these volitions do not change—because "God necessarily loves the eternal arche-

* "Que vous exauciez cette prière, après que vous l'aurez *formée* en moi!"—*Méditations*, v.

typal forms which are included in His own substance." Nothing could be farther from our author than to symbolize with certain wild speculations of a portion of the Neo-Platonic school of Alexandria, to the effect that "God himself would have been more perfect, if imperfect creatures had not received existence from His power:" yet, in one of his Meditations, he singularly speaks of the Deity as "condescending to assume the low and humiliating character of *creator*."*

Nothing can be more excellent than the practical moral tone which pervades all the writings of our philosopher; who, in reference to his speculative theories, was named by some of his opponent contemporaries the "Dreamer of the Oratory."† At all events, his dreams are pure, devout, and elevated—contrasted enough with the mass of visionary matter contained in the novels and romances which are always issuing from the European press. We wish that our educated youth of both sexes who desire to exercise themselves in French reading, would procure the two small and portable volumes of M. Simon's edition of Malebranche, as a substitute for the trash which is sometimes devoured on the plea of improvement in the language. The charms of the style, the ingenuity, the animation, and the subject itself, would amply repay the perusal; and though, as in all other books which aim at solving the mysteries of God, in nature, providence, and grace, and framing them into a system, the philosophy breaks down—much, very much remains that is eminently calculated to benefit the understanding and the heart. The excellent qualities of the author's mind are everywhere patent, and never perhaps was the saying *Le style c'est l'homme* better illustrated than by Malebranche.

Whatever inferences might be consequently drawn from any of his dogmas, it is certain that neither his own practice nor his general views of human nature and duty were injuriously affected by them. The grandeur, piety, and justness of his conceptions break forth even when he is treating of his theory of occasional causes, against which so much objection has always been made. After having laboured to show that the will of God alone is efficacious, and that it is His power which produces all effects, he exclaims: "O Theodorus! O Theotimus! God alone is the bond of our social union! Let Him then be the great end, since he is the beginning. Let us not abuse His power. Woe to them who make that power the pander to their passions! Nothing is more sacred than power; nothing is more

* "Méditations," xix. 5.

† An epigrammatic line was current: "Lui qui voit tout en Dieu, n'y voit-il pas qu'il est fou?" La Harpe adds: "C'était au moins un fou qui avait beaucoup d'esprit."

divine. It is a species of sacrilege to make a profane use of power. I see, to a demonstration, that it is to render the just avenger of crimes a slave to iniquity! Of ourselves we can do nothing, therefore we ought of ourselves to will nothing. We can only act by the efficacy of the Divine power, therefore we ought to will nothing but what is in accordance with the Divine law. Nothing is more evident than these truths. The law of duty is the foundation of all morals. Holy law! which Christians call the love of God, because their God being goodness itself, to obey duty, to love duty, is to obey God, and to love Him above all creatures. We should never love any good absolutely, if it is possible for us not to love it without remorse."*

It is remarkable (and it ought to be a motive for candid judgment) how often we find men shrinking, with a degree of horror, from what appear to others to be the legitimate consequences of their principles when logically and in detail carried out. The whole *tendency* of the more abstract and theoretic part of the philosophy of Malebranche was unquestionably to merge all second causes in the agency of the one, first, infinite cause; and in like manner to absorb all created being in the infinite and the absolute—especially to sink the very perception and consciousness of man in a mysterious union with the Deity, in whom all was seen, and known, and done. Malebranche, in fact, conducted the Cartesians to the threshold of Spinozism. Yet nothing was more painful to his mind than those criticisms of his contemporaries which endeavoured to establish analogies between his system and that of Spinoza—"that miserable and impious man," as he always termed him. Mairan, who had been reading the "Ethica" of Spinoza, pointed out to Malebranche the similarity of many of its statements to those of his own "Recherche;" but he positively refused to discuss the subject. In one of his dialogues, in evident allusion to the dogmas of Spinoza respecting the Divine Being, and with little disposition to exercise any gratuitous candour towards that writer, he makes Theodorus say to Aristus: "What a monster, Aristus!—a God necessarily hated, blasphemed, despised! Assuredly, if there are people capable of inventing such a God, upon ideas so monstrous, it is either that they wish to have no God, or else such spirits are born to seek, in the idea of the circle, all the properties of triangles."†

While the reputation of our author, as a thinker and writer, has survived him without interruption to the present time, especially in his own country; one reason why he did not very long retain his influence as a leader in philosophy was, that he was eclipsed by Leibnitz in Germany, and by Locke in England and France.

* "Entretiens sur la Métaphysique," vii.

† *Ibid.* ix.

His works, however, can never cease to attract readers, so long as the French language lasts. He addresses himself, with Descartes, to the philosopher, and with Pascal and Fénélon to the devout; he is the metaphysician, the moralist, and the theologian; and those parts of his writings, especially, which are penned in the dialogue-form, have never been surpassed in brilliancy, point, and life, by any similar production on subjects mainly addressed to the understanding, and cannot fail to prove both instructive and interesting to all readers whose object is improvement.

